# Achieving Giant Piezoelectricity and High Property Uniformity Simultaneously in a Relaxor Ferroelectric Crystal through Rare‐Earth Element Doping

**DOI:** 10.1002/advs.202204631

**Published:** 2022-10-26

**Authors:** Yangbin Liu, Qian Li, Liao Qiao, Zhuo Xu, Fei Li

**Affiliations:** ^1^ Electronic Materials Research Laboratory Key Laboratory of the Ministry of Education & International Center for Dielectric Research School of Electronic and Information Engineering Xi'an Jiaotong University Xi'an 710049 China

**Keywords:** piezoelectricity, rare‐earth doping, relaxor ferroelectric crystals, single‐crystal growth

## Abstract

The low uniformity in properties of relaxor ferroelectric crystals is a long‐standing issue in the ferroelectric community, which limits the available volume of the entire crystal boule. The aim of this study is to develop a relaxor ferroelectric crystal with improved property uniformity and excellent piezoelectricity. To this end, Pb(In_1/2_Nb_1/2_)O_3_–Pb(Mg_1/3_Nb_2/3_)O_3_–PbTiO_3_ is doped with Nd_2_O_3_ (Nd–PIN–PMN–PT) to improve the crystal performance. Along the crystal boule, the piezoelectric coefficient *d*_33_ varies from 2800 to 3500 pC N^−1^, and the dielectric constant ranges from 8400 to 9800, with variations of 25% and 16%, respectively. Such high property uniformity results in over 75% available volume of the crystal boule, compared to 30–50% for undoped crystals grown by Bridgman method. At the electric field of 1 kV cm^−1^, the converse piezoelectric response is up to 4780 pm V^−1^. In addition, its Curie temperature (*T*_C_) and coercive field (*E*_C_) are above 150 °C and 3 kV cm^−1^, respectively. Compared with Pb(Mg_1/3_Nb_2/3_)O_3_–PbTiO_3_ crystal (*d*_33_: 1500 pC N^−1^, *T*_C_: 135 °C, *E*_C_: 2.3 kV cm^−1^), the larger piezoelectricity, the higher *T*_C_ and *E*_C_, and improved uniformity make Nd–PIN–PMN–PT crystals promising candidates for advanced piezoelectric applications.

## Graphical abstract

An Nd‐doped Pb(In_1/2_Nb_1/2_)O_3_–Pb(Mg_1/3_Nb_2/3_)O_3_–PbTiO_3_ relaxor ferroelectric crystal with piezoelectric coefficient *d*_33_ up to 3500 pC N^−1^ is successfully grown by Bridgman method. Compared with commercial Pb(Mg_1/3_Nb_2/3_)O_3_–PbTiO_3_ crystals, the larger piezoelectricity, higher *T*c and *E*_C_, and improved property uniformity are observed in Nd‐doped PIN–PMN–PT crystals, which make them promising candidates for advanced piezoelectric applications.

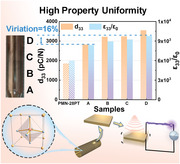

## 1 Introduction

Since the piezoelectricity of relaxor ferroelectric solid‐solution single crystals was characterized for the first time in 1982,^[^ ^1^ ^]^ they have attracted much attention in the field of ferroelectric materials. For example, Pb(Mg_1/3_Nb_2/3_)O_3_–PbTiO_3_ (PMN–PT) and Pb(Zn_1/3_Nb_2/3_)O_3_–PbTiO_3_ (PZN–PT) crystals have excellent piezoelectric properties (piezoelectric coefficient *d*_33_ = 1200–2500 pC N^−1^, electromechanical cuopling factor *k*_33_ > 90%), which are far superior to those of piezoelectric ceramics.^[^ ^2^, ^3^, ^4^, ^5^ ^]^ Relaxor ferroelectric single crystals have been applied to high‐performance electromechanical applications, especially high‐frequency and low‐driven‐field transducers.^[^ ^6^, ^7^, ^8^, ^9^, ^10^ ^]^ In 2019, Li et al. fabricated Sm‐doped PMN–PT(Sm–PMN–PT) single crystals that showed much improved performance compared to that of the existing relaxor ferroelectric single crystals; for example, these single crystals achieved a giant piezoelectric coefficient *d*_33_ of ≈4000 pC N^−1^.^[^ ^11^ ^]^ These findings led to further research on improving the piezoelectric performance by modifying the local heterogeneity microscopically.^[^ ^12^, ^13^ ^]^

Although relaxor ferroelectric single crystals have excellent piezoelectric and dielectric properties, there are still challenges to be overcome. The first is the uniformity of the entire single crystal. It is well known that relaxor ferroelectric single crystals with high performance are almost all binary or ternary solid solutions such as PMN–PT, PZN–PT, and Pb(In_1/2_Nb_1/2_)O_3_–Pb(Mg_1/3_Nb_2/3_)O_3_–PbTiO_3_ (PIN–PMN–PT).^[^ ^6^, ^7^ ^]^ To obtain large samples, ferroelectric single crystals are generally grown using the Bridgman method.^[^ ^14^ ^]^ The segregation during growth leads to inhomogeneous composition (and hence, properties) along the growth direction of the single crystal boule. The resulting piezoelectric and dielectric properties of such single crystals usually vary over a wide range. Therefore, the usable volume of the single crystal is only a small fraction of the total volume (≈50%),^[^ ^7^, ^11^ ^]^ which results in a high cost of material manufacturing. In addition, the inhomogeneous composition and properties across individual samples pose problems for device development. Such inhomogeneity induced by segregation during processing has been a long‐standing bottleneck in the field. Therefore, researchers have developed various methods for suppressing segregation. For example, better property uniformity can be achieved with the continuous‐feeding Bridgman method than that with the conventional Bridgman method.^[^ ^15^, ^16^, ^17^ ^]^ In the modified process, a feeding mechanism continuously feeds the raw material into the crucible during crystal growth to obtain homogeneous piezoelectric properties along the crystal boule. The flux method and solid‐solution single crystal growth were also used to produce ferroelectric single crystals with high property uniformity.^[^ ^18^, ^19^, ^20^, ^21^, ^22^, ^23^ ^]^ However, these methods have limitations. The continuous‐feeding Bridgman method increases the complexity and cost of the growth process compared to that of the conventional method, while the flux and solid‐solution single crystal growth methods cannot achieve single crystals with large sizes, for example, >3 inches.

Another long‐standing challenge is overcoming the trade‐off between the phase‐transition temperature and piezoelectric performance. Generally, enhancements of the piezoelectric performance are always gained at the sacrifice of the phase‐transition temperature or coercive field (*E*_C_), and vice versa. Generally, it is difficult to enhance them simultaneously through methods such as doping and alternating current (AC) poling.^[^ ^11^, ^14^ ^]^ The phase‐transition temperature and *E*_C_ are important because they limit the operating temperature and drive the electric field of the piezoelectric devices. Specifically, when the temperature exceeds the phase‐transition temperature or the applied electric field exceeds *E*_C_, the piezoelectric performance of the material is impaired. Thus, depending on the final application, researchers generally select the parameter they want to optimize while sacrificing other properties. Rare‐earth elements have been used to achieve giant piezoelectric performance by flatting the free energy profile. However, these crystals suffer from a low rhombohedral–tetragonal phase‐transition temperature (*T*_RT_ ≈61 °C).^[^ ^11^ ^]^ On the contrary, Mn is doped into PMN–PT single crystal to introduce oxygen vacancy and achieve higher *E*_C_ at the expense of piezoelectric performance.^[^ ^24^ ^]^ In addition, although the state‐of‐the‐art method of AC poling can be used to control domain structure for enhancing piezoelectric performance, it also reduces *E*_C_.^[^ ^14^ ^]^ Thus, these strategies indeed help to overcome some problems, but further development in piezoelectric devices requires materials with both ultra‐high piezoelectric properties and high phase‐transition temperature and *E*_C_.

In this study, the rare‐earth element Nd was introduced into PIN–PMN–PT crystals in an attempt to achieve both giant piezoelectric performance and improved property uniformity. The Nd^3+^ dopant flattens the Gibbs free energy density profile according to Landau phenomenological theory.^[^ ^25^, ^26^, ^27^, ^28^ ^]^ On the microscopic scale, it enhances the fluctuation in the crystal lattice and increases the quantity of polar nano‐regions (PNRs) to enhance the piezoelectric performance.^[^ ^29^, ^30^, ^31^, ^32^ ^]^ Here, we demonstrate the successful fabrication of an Nd–PIN–PMN–PT single crystal and provide a strategy to improve property homogeneity, electric performance, and stability simultaneously by Nd doping.

## 2 Results and Discussion

### 2.1 Giant Piezoelectricity and High Property Uniformity

**Figure 1:**
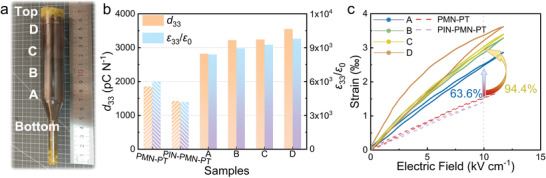
a) Photograph of an as‐grown Nd–PIN–PMN–PT single crystal. Samples A–D was cut every 20 mm to measure the b) piezoelectric coefficient *d*_33_ and dielectric permittivity *ε*_33_/*ε*_0_ properties, and c) unipolar strain along the crystal boule. The results are compared to PMN–28PT and PIN‐PMN‐PT single crystal.

A photograph of a grown Nd–PIN–PMN–PT single crystal boule obtained by the modified Bridgman method is shown in **Figure** **1**a. The boule had a diameter of 25.4 mm and was over 90 mm in length for the cylindrical part. In this work, we aimed to suppress the property inhomogeneity and enhance the piezoelectric performance along the single crystal boule by the introduction of Nd^3+^ ions. Both piezoelectric and inverse piezoelectric effects are applied to various acoustic applications such as underwater acoustic devices, medical ultrasound imaging, ultrasonic stone crushing, and wearable equipment for energy harvesting or health monitoring. Figure 1b shows that the *ε*_33_/*ε*_0_ and *d*_33_ values of all samples exceeded 8000 and 2700 pC N^−1^, respectively. Samples B–D (at least 20 mm from the bottom of the boule) showed better performance than sample A, with *d*_33_ > 3000 pC N^−1^. These values were much higher than those for a mature commercial ferroelectric single crystal (PMN–28PT and PIN–PMN–PT).^[^ ^14^ ^]^ A similar comparison is shown in Figure 1c for the inverse piezoelectric effect; the strains of all Nd–PIN–PMN–PT samples were much higher than those of PMN–28PT and PIN–PMN–PT. Among the samples, the strain of specimen D had the largest hysteresis, which might cause large loss and signal delay under a large electric field during operation. This hysteresis is derived from an electric‐field‐induced phase transiti. For confirming this, the strain of sample D under different unipolar electric fields was measured and plotted in Figure S1d, Supporting Information. When the applied electric field is below 8 kV cm^−1^, the unipolar strain exhibits low hysteresis (at 2 and 5 kV cm^−1^). This result also proved that the large hysteresis of strain at 11 kV cm^−1^ originated from phase transition. Roughly stated, under a high electric field, the phase structure transforms from a rhombohedral phase to a tetragonal or orthorhombic phase for minimizing the free energy of the crystal. Apart from sample D, the other samples showed little hysteresis. When measured at 10 kV cm^−1^, the strain of sample A exceeded those of PMN–P28T and PIN–PMN–31PT by more than 60%, while that of sample C was over 94% higher than those of PMN–PT and PIN–PMN–PT. In conclusion, Nd–PIN–PMN–PT single crystals showed better piezoelectric performance than PMN–PT and PIN–PMN–PT. It could make great contributions to piezoelectric devices with improved performance.

Furthermore, it can be seen from Figure 1b,c that the Nd–PIN–PMN–PT single crystal had excellent uniformity along the axis of the crystal boule. Figure 1b shows that both *ε*_33_/*ε*_0_ and *d*_33_ remained stable along the crystal boule. Specifically, the variation in *ε*_33_/*ε*_0_ was 16.7% (8400 for sample A to 9800 for sample D) and for *d*_33_ it was 25.8% (2820 to 3550 for sample A to D, respectively). In addition, the strain had low variation (29% across the boule) at 10 kV cm^−1^. In order to fully reveal the property uniformity of the crystal boule, *d*_33_ and *ε*_33_/*ε*_0_ along the radial direction were also measured and plotted in Figure S2, Supporting Information, and the low variation (<5%) of *d*_33_ and *ε*_33_/*ε*_0_ indicated our crystal also possessed high uniformity along the radial direction. Overall, all of the important parameters varied by <30% along the boule. Conversely, the properties of undoped PIN–PMN–PT single crystals obtained by the conventional Bridgman method varied by a greater degree;^[^ ^33^ ^]^ *d*_33_ varied by over 100% along the single crystal boule (1000–2035 pC N^−1^), and *ε*_33_/*ε*_0_ varied by 68% (3200 to 5391). The tetragonal phase part at the top of the crystal boule further reduces the availability of volume. Such large variations in properties are one of the most important reasons why relaxor ferroelectric single crystals have not yet replaced piezoelectric ceramics. In addition, the low utilization ratio of single‐crystal boules results in a high final cost of application, which further limits the development of ferroelectric single‐crystal devices. In previous research, the useable volume of PMN–PT and PIN–PMN–PT single‐crystal boules was typically only 30% to 50%.^[^ ^7^ ^]^ In the case of our Nd–PIN–PMN–PT single crystal, the low variation in piezoelectric and dielectric properties increases the useable volume (high *d*_33_ and low hysteresis) to over 75%, which will reduce the crystal cost. If the target application requires low voltage, the entire crystal boule can be fully utilized. Therefore, the introduction of Nd ions apparently improved the homogeneity of the properties along the single‐crystal boule.

### 2.2 Compositional Segregation and Phase Structure

**Figure 2:**
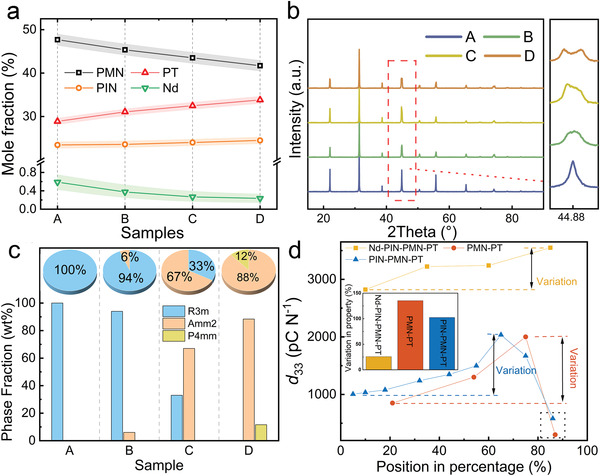
a) Mole fraction of each component to elucidate the segregation of PIN, PMN, PT, and Nd^3+^ ions along the boule. The shaded regions around the data indicate the experimental error. b) XRD patterns of samples A–D (ground into powder). Inset: the enlarged region around 2*θ* = 45°. c) The weight fraction of different phase structures (or space groups) for all samples. d) The contrast of uniformity among Nd‐doped PIN‐PMN‐PT, PMN‐PT, and PIN‐PMN‐PT single crystals obtained by the conventional Bridgman method.

The inhomogeneity of solid‐solution single crystals is mainly induced by segregation during crystal growth. Therefore, the high property uniformity of our Nd–PIN–PMN–PT single crystal could be explained by the structure and composition of the samples obtained along the boule. X‐ray diffraction (XRD) and electron probe microanalysis (EPMA) were performed for samples A–D. **Figure** **2**a shows the mole fraction of each component in Nd–PIN–PMN–PT along the single crystal to quantify the segregation effect. The content of component PMN clearly decreased from the bottom (sample A) to the top (sample D) of the crystal boule, while the content of component PT showed the opposite trend. In contrast to components PMN and PT, PIN did not segregate significantly along the growth direction, which is consistent with previously published results.^[^ ^33^, ^34^ ^]^ Furthermore, the dopant Nd^3+^ ions segregated during single‐crystal growth, and their fraction decreased along the single‐crystal boule with a similar trend to PMN and an opposite trend to PT. The *d*_33_ and *ε*_33_/*ε*_0_ of crystals with rhombohedral phase will undoubtedly increase while the content of component PT increases. Thus, quite a large volume at the bottom of the single‐crystal boule usually cannot be used because of its low PT content (and accordingly low piezoelectric performance). This greatly contributes to the high cost of ferroelectric single crystals. Fortunately, the introduction of rare‐earth dopant Nd changes the situation. The Nd^3+^ ions substituted A‐site Pb^2+^ ions and accordingly enhanced the piezoelectric and dielectric properties. Both Nd substitution and PT favor the piezoelectric performance. Their opposite segregation trends led to high uniformity in the properties along the entire crystal boule, which gave an equivalent effect to that of segregation being suppressed. To verify the segregation, powder XRD patterns of all samples were obtained (Figure 2b) and a pure perovskite structure was observed, as expected. The inset shows a magnified view of the peaks around 45° to provide details of the patterns. The symmetric peak at ≈45° for samples A and B implies that they possess a rhombohedral phase. In contrast, the asymmetry in the equivalent peak for sample C implies that the structure is near the morphotropic phase boundary (MPB). The XRD pattern of sample D had two distinct peak profiles, but they were not split completely. This indicates that sample D is in the orthorhombic phase or mixed phase of orthorhombic and tetragonal. These phase differences among samples A–D revealed differences in their lattice parameters. To verify it, the Rietveld method was used in the refinement to carry out the lattice parameters and the phase structure of all samples. The detailed refinement results are given in Figure S3, Supporting Information. The weight fractions of different phase structures for all samples are plotted in Figure 2c. Unlike sample A, which possesses a pure trigonal phase (Space group: R3m), samples B to D are in a mixed phase with multiple phase structures. The main phase structure of sample B is the rhombohedral phase, while those of samples C and D are orthorhombic phases (Space group: Amm2). As PT content increases, the structure of Nd‐doped single crystal transfers from the rhombohedral phase to the orthorhombic phase gradually. The very minor volume of the tetragonal phase at the top of the crystal boule (sample D) also indicates that the variation of microstructure is reduced by Nd‐doping. The reason why the uniformity of structure was also improved is that the segregation law of Nd atoms in the crystal is opposite to that of PT, as shown in Figure 2a. In addition, the phase structure of the crystal will approach MPB and tetragonal phase as the contents of rare‐earth dopant and PT increase. Figure 2d shows that undoped PMN–PT and PIN–PMN–PT single crystals typically enter the tetragonal phase at the position of ≈60–80% from the bottom of the crystal boule, while Nd‐doped single crystal does not show a tetragonal phase even at the top of the crystal. The lattice parameters of all samples are listed in Table S1, Supporting Information. The XRD patterns of all specimens as a function of temperature (30–190 °C) are shown in Figure S4, Supporting Information. These results show that all samples underwent two phase transitions, that is, ferroelectric‐to‐ferroelectric and ferroelectric‐to‐paraelectric phase transitions.

### 2.3 PNR Activation at Cryogenic Temperature

**Figure 3:**
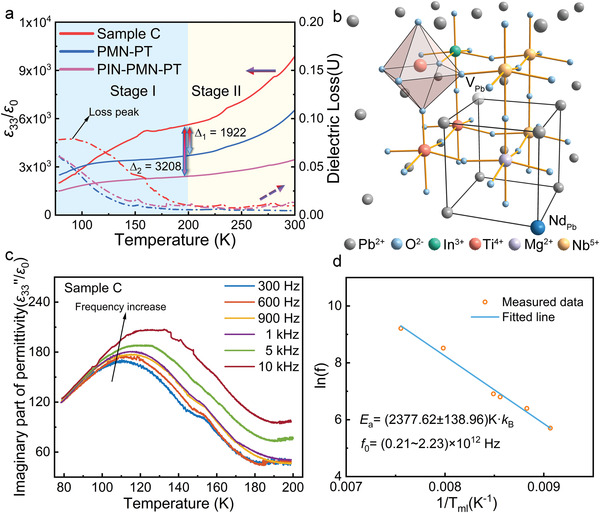
a) Dielectric permittivity *ε*_33/_ *ε*_0_ and dielectric loss of Nd–PIN–PMN–PT (sample C), PMN–28PT, and PIN‐PMN‐31PT at cryogenic temperatures. The temperature range (80–320 K) is split into two stages for discussion. b) Illustration of the crystal structure of the Nd–PIN–PMN–PT single crystal showing how Nd^3+^ ions substitute for Pb^2+^ ions and the formation of a Pb vacancy. c) The temperature dependence of the imaginary part of dielectric permittivity (*ε*_33″_/*ε*_0_) and d) the frequency dependence of *T*_ml_ were measured for the low‐temperature relaxation characteristic.

The high piezoelectricity of relaxor‐PT ferroelectric single crystal was considered to originate from the PNRs. The presence of PNRs in relaxor ferroelectrics has been confirmed experimentally,^[^ ^8^, ^9^ ^]^ and they greatly contribute to the piezoelectric and dielectric properties. Specifically, the PNRs act as “seeds” at room temperature, which facilitate the polarization rotation process under an applied electric field.^[^ ^35^ ^]^ In addition, one of the typical features of PNRs is the increased dielectric loss and frequency diffusion of dielectric permittivity at cryogenic temperature. To investigate the role of Nd^3+^ ions in PNRs, *ε*_33_/*ε*_0_ at cryogenic temperature (cooled to 80 K using liquid nitrogen) was characterized. As illustrated in **Figure** **3**b, when Nd^3+^ ions (dark blue sphere) entered the lattice of the ABO_3_ perovskite, they substitute for Pb^2+^ ions (grey sphere) and formed lead vacancies (translucent sphere in the center). The smaller ionic radius of Nd^3+^ (1.27 Å) than that of Pb^2+^ (1.49 Å) and the presence of vacancies result in severe lattice distortion, leading to the additional fluctuation in the crystal lattice and the formation of PNRs. To confirm the effect of PNRs in Nd–PIN–PMN–PT, we measured *ε*_33_/*ε*_0_ at cryogenic temperature for sample C, and compared the data to that for PMN–28PT and PIN–PMN–PT as a reference, as shown in Figure 3a. The measured temperature range (80–320 K) was classified into two parts for discussion: stage I (below 200 K) and stage II (above 200 K). The temperature of 200 K was chosen because the curves of dielectric loss below and above 200 K were different, which represented different states of PNRs. In detail, the curves for both PMN–28PT and sample C have obvious broad dielectric loss peaks in stage I. Usually, dielectric loss anomaly peaks are related to phase transition, domain switching, or interface motion.^[^ ^11^, ^36^, ^37^ ^]^ Our previous work proved by XRD that the dielectric loss peak at cryogenic temperature did not originate from phase transition and was instead associated with the switching of polar vectors or interface motion of local heterogeneous regions.^[^ ^38^ ^]^ In stage I, the *ε*_33_/*ε*_0_ of Nd‐doped single crystals increased significantly with increasing temperature. It was confirmed that the increase in dielectric permittivity at cryogenic temperature was related to the switch of PNRs. Therefore, as the temperature increased in stage I, polar vectors of partial PNRs began to switch to the long‐range ordered domains from their original direction. After switching to stage II (*T* > 200 K), these PNRs became collinear with the long‐range ordered domains and were in the same direction. However, they were easier to switch under an electric field compared with long‐range ordered ferroelectric domains. It can be seen that the temperature‐dependent dielectric loss of PMN–PT and PIN–PMN–PT share a similar trend in both stage I and stage II, which is different from that of the Nd–PIN–PMN–PT single crystal. Since the anomaly dielectric behavior at a cryogenic temperature of relaxor PT single crystal is mainly affected by the presence of PNRs, the difference in dielectric loss between Nd‐doped PIN–PMN–PT single crystal and undoped crystals was mainly caused by PNRs instead of the long‐range ferroelectric domains. At temperatures below 100 K, the dielectric permittivity of Nd–PIN–PMN–PT was lower than that of PMN–PT. However, at temperatures above 110 K, the dielectric permittivity of Nd–PIN–PMN–PT exceeded that of PMN–PT. The dielectric permittivity of Nd–PIN–PMN–PT is larger than that of PIN–PMN–PT in the temperature range of 80 to 300 K, which indicated a larger piezoelectric *d*_33_ when compared to the undoped PIN–PMN–PT. In addition, at 200 K the dielectric permittivity of Nd‐doped PIN–PMN–PT (nearly 6000) was about 1920 higher than that of PMN–PT, and 3200 higher than that of PIN–PMN–PT.

Since the dielectric anomaly behavior at cryogenic temperatures is a typical feature of relaxation related to PNRs. It should be paid attention that in stage I, the increase in the dielectric constant of Nd–PIN–PMN–PT was significantly greater than those of PMN–PT and PIN–PMN–PT with increasing temperature. To investigate the role of PNRs at cryogenic temperature, the dielectric permittivity at cryogenic temperature was measured at different frequencies. Arrhenius law was employed to study the relationship between the temperature (*T*_ml_) of maximum permittivity *ε*_33_’’/*ε*_0_ and the frequency (Figure 3c)

(1) $$f=f_{0}\exp\left[-E_{a}/k_{B}T_{\mathrm{ml}}\right]$$

where *f* is the measurement frequency, *E*_a_ is the activation energy, *k*_B_ is the Boltzmann constant, *f*_0_ is the attempt frequency, and *T*_ml_ is the temperature of maximum *ε*_33_’’/*ε*_0_ (imaginary dielectric permittivity) at low temperature. It can be observed that ln(*f*) and 1/*T*_ml_ follow a linear relationship as shown in Figure 3d, which agreed with the Arrhenius law. The activation energy *E*_a_ and attempt frequency *f*_0_ were gotten from the fitted line. The activation energy *E*_a_ of Nd–PIN–PMN–PT is about 2378 K × *k*_B_, which has a similar level to that observed in PMN,^[^ ^45^ ^]^ being related to the switching energy barrier of PNRs. Therefore, the increase of dielectric permittivity with an increasing temperature of Nd–PIN–PMN–PT single crystal was considered to be associated with the presence of PNRs.

### 2.4 Contributions to Piezoelectric Performance

For domain‐engineered crystals, there are many factors that can affect the piezoelectric response such as intrinsic contributions and movement of the interface. The various contributions to the high piezoelectric performance of the Nd–PIN–PMN–PT single crystal were analyzed by Rayleigh's Law.^[^ ^39^, ^40^ ^]^ For piezoelectric materials, Rayleigh's Law is expressed as

(2) $$S\left(E\right)=\left(d_{\mathrm{init}}+E_{0}\alpha \right)E\pm \frac{\alpha }{2}\left(E_{0}^{2}-E^{2}\right)$$

(3) $$d\left(E_{0}\right)=\left(d_{\mathrm{init}}+E_{0}\alpha \right){\mathrm{pC}N}^{-1}$$

Here, *S*(E) is the strain under the electric field; *E*_0_ is the amplitude of the applied electric field, and *d*_init_ is the Rayleigh coefficient. The *d*_init_ parameter describes the reversible piezoelectric response associated with the intrinsic contribution and the contribution of the reversible motion of the internal interface (domain wall and phase boundary). In reversible motion, the internal interface recovers to its original state after removing the applied electric field. Considering [001] engineered domains with rhombohedral or orthorhombic phase (4R or 4O, respectively), the four energetically equivalent domains and stable domain configuration minimize the contribution of the reversible domain wall motion. This is because all engineered domains in [001] poled rhombohedral crystals are equivalent along the direction of the electric field [001], and switching among these domains do not contribute to the piezoelectric response along [001]. Thus, here we regard *d*_init_ as the intrinsic contribution from the lattice. Another coefficient *α* represents the irreversible motion of the internal interface, for example, the interface between rhombohedral and some minor monoclinic phase regions. Specifically, when the applied electric field is removed, the internal interface cannot catch up with the change of the electric field and return to its original state in time, resulting in the hysteresis observed in electric‐field‐induced strain curves. In addition, the external contribution is given as *E*_0_ *α*. In Equation (2), “+” and “−” correspond to the decreasing and increasing electric field, respectively. Here, *d*_init_ and *α* were obtained by fitting our experimental data with Equation (3), and the effectiveness of the obtained *d*_init_ and *α* was also verified by Equation (2).

**Figure 4:**
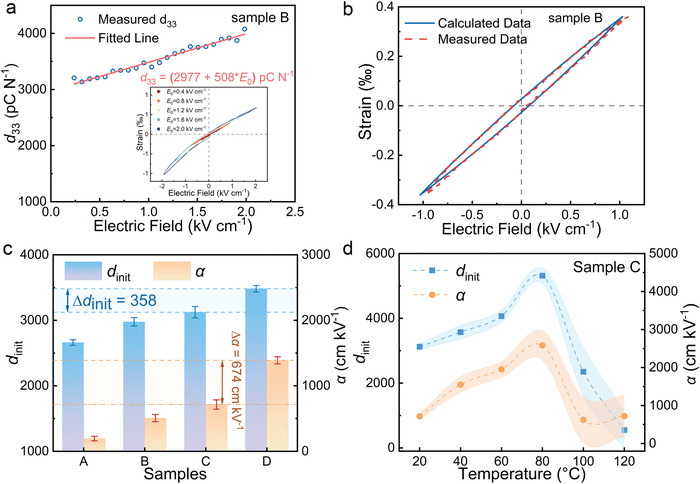
a) AC electric‐field‐dependent *d*_33_ and the b) experimental and calculated strain of sample B. c) Fitted Rayleigh coefficients *d*_init_ and *α* for the different samples along the Nd‐doped single crystal. d) Rayleigh coefficients of sample C as a function of temperature; shaded regions are error bars.

**Figure** **4**a shows the experimental *d*_33_ data and the fitted line by Equation (2) to obtain the *d*_init_ and *α* of sample B. The calculated and experimental data of strain versus electric field coincided well, as shown in Figure 4b. This implies that the Nd–PIN–PMN–PT samples obeyed Rayleigh's Law. The *d*_init_ and *α* of the other samples were derived, as shown in Figure S6, Supporting Information. All obtained coefficients are plotted in Figure 4c. Both *d_i_*_nit_ and *α* increased along the single crystal boule as the PT content increased. Moreover, *α* of sample D showed an increase in the figure, which indicated a domain structure around the MPB. The difference in *α* between sample C and sample D (674 cm kV^−1^) was even larger than that between samples A and C (520 cm kV^−1^). Compared with PMN–PT,^[^ ^41^ ^]^ our Nd‐doped single crystal had both higher *d*_init_ and *α*. Therefore, lattice distortion due to Nd substitution and Pb vacancies affected the intrinsic contribution, and also promoted irreversible domain wall motion. The ratios of the intrinsic contribution to the total piezoelectric response *d*_init_/(*E*_0_ *α* + *d*_init_) are shown in **Table** **1**. These values decreased as the content of PT increased. The Nd substitution promoted the formation of PNRs, where these regions usually have higher local system energy compared to the long‐range‐ordered domain structure. In this case, when an electric field is applied, PNRs more easily switch than the long‐range‐ordered domains. The difference in polarization switching between PNRs and the long‐range‐ordered domains resulted in the motion of the interface between them, which can drive the entire domain structure into another potential well (irreversible interface motion). In addition, when the phase structure of the single crystal was close to the MPB (high PT content), there may be two or more phases in the local structure owing to compositional fluctuations.^[^ ^41^ ^]^ The presence of PNRs aggravates this situation. Moreover, PNRs with the high system free energy act as a seed to help adjacent domains switch or even to transform to another phase, which explains why *α* increases greatly at the position of sample D (near MPB). The Rayleigh coefficients of sample C were determined at different temperatures, as shown in Figure 4d, Supporting Information; both *d*_init_ and *α* first increased and then decreased with increasing temperature. At 100 °C, both *d*_init_ and *α* were low, which is consistent with the piezoelectric properties of the tetragonal phase. In addition, the value of *α* shows an increasing trend at 120 °C owing to the tetragonal phase structure.

**Table 1:** Calculated Rayleigh coefficients of Nd–PIN–PMN–PT single crystals

Sample	*α* [cm kV^−1^]	*d*_init_	*E*_0_ *α*+ *d*_init_ [pC N^−1^]	*d*_init_/(*E*_0_ *α*+ *d*_init_) [%]
A	194	2661	2855	93.2
B	508	2977	3485	85.4
C	715	3124	3839	81.4
D	1389	3482	4871	71.5

### 2.5 Stability

**Figure 5:**
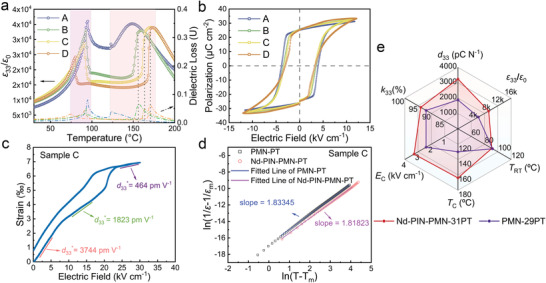
a) Temperature‐dependent dielectric permittivity of specimens of all samples. Ferroelectric properties: b) hysteresis loops of samples A–D, and c) strain of sample C under a unipolar electric field with high amplitude. d) log(1/*ε* − 1/*ε*_m_) versus log(*T* − *T*_m_) curves at temperatures above *T*_m_ calculated from Curie–Weiss law, at 1 kHz. The slope represented the relaxation level. e) Radar chart comparing the overall performance of Nd–PIN–PMN–PT with published PMN–29PT single crystals.^[^ ^24^ ^]^

In piezoelectric transducer fabrication, if the fabrication temperature exceeds the Curie temperature (*T*_C_), the transducers will no longer operate due to depoling. If the operating temperature exceeds *T*_RT_ or the operating electric field exceeds *E*_C_, the transducer becomes impaired or even destroyed. Therefore, the piezoelectric performance should be stable over a range of different temperatures and electric fields to facilitate the development and operation of piezoelectric devices. Thus, the temperature‐dependent dielectric permittivity and hysteresis loop of as‐grown Nd–PIN–PMN–PT single crystals were measured and plotted in **Figures** **5**a and 5b, respectively. All samples of Nd–PIN–PMN–PT single crystal possess two obvious dielectric anomaly peaks. For sample A in Figure 5a, the first sharp peak at 95 °C corresponds to the rhombohedral‐tetragonal phase transition temperature *T*_RT_. The second dielectric peak *T*_m_ at 148 °C of sample A is quite broad, being related to the relaxor feature. For samples B, C, and D, the second dielectric peak is the temperature of macro‐micro ferroelectric domain transition, in which long‐range ferroelectric domains are broken and the crystals are depoled. Thus, this temperature is generally called as depolarization temperature *T*_d_. In general, with the increase in PT content (samples A to D), the peak related to *T*_C_ (the second peak in dielectric loss) was observed at a higher temperature. The distribution of the *T*_C_ values was wide, from 123 to 170 °C (light pink region). In contrast, as the PT content increased, the *T*_RT_ shifted to a lower temperature. These peaks were narrowly distributed, from 80 to 95 °C (light purple region). Figure 5b shows the entire hysteresis loops of different samples along the crystal boule. The *E*_C_ values were all above 3 kV cm^−1^ and remained relatively stable as the PT content increased (samples A to D), while the remnant polarization increased slightly. All samples (samples A to D) had higher *E*_C_ values than that of PMN–PT, implying that transducers made from Nd–PIN–PMN–PT single crystal could withstand a higher electric field than PMN–28PT during operation. Among all samples, sample C had simultaneously high piezoelectric performance, high phase transition temperature, and small strain hysteresis. Thus, sample C was chosen to test the piezoelectric and ferroelectric properties under different conditions. In Figure 5c, the strain of sample C under a unipolar electric field is shown. The nonlinear part in the strain curve with large hysteresis indicates that the crystal is undergoing a phase transition. In Figure 5c, as the applied electric field increased to 35 kV cm^−1^, sample C underwent two electric‐field‐induced phase transitions. Sample C maintained its original phase structure below 9 kV cm^−1^, and then underwent two phase transitions at the electric fields of 11 and 22 kV cm^−1^, respectively. According to the previous works,^[^ ^41^, ^42^ ^]^ the phase structure of sample C at the E‐field of 13–20 kV cm^−1^ is inferred to be a monoclinic phase. When the applied electric field is above 24 kV cm^−1^, sample C was transformed to the tetragonal phase because the [001] electric field, which is along the polar direction of the tetragonal phase, favors the tetragonal phase.

As a relaxor ferroelectric single crystal, the relaxor behavior of sample C of Nd–PIN–PMN–PT single crystal was evaluated and analyzed based on the modified Curie–Weiss law

(4) $$\frac{1}{\varepsilon }-\frac{1}{\varepsilon_{m}}=\frac{{\left(T-T_{m}\right)}^{\gamma }}{C}$$

where *ε*_m_ is the maximum value of the dielectric permittivity at *T*_m_, *C* is the Curie‐like constant, and *γ* is the degree of diffuseness (or relaxor factor). A higher value of *γ* indicates a higher level of relaxor behavior. For classical ferroelectrics, *γ* is 1, while it is 2 for relaxor ferroelectrics; *γ* corresponds to the slope of the fitted line calculated by Equation (4) and temperature‐dependent dielectric permittivity. Figure 5d shows that sample C and PMN–28PT had similar *γ* values of 1.81 and 1.83, respectively, indicating similar relaxor behaviors for these two single crystals.

For comparison, the overall properties of sample C and published PMN–29PT single crystals are plotted together in Figure 5e, and the properties of more single crystals are listed in **Table** **2**.^[^ ^11^, ^24^, ^33^, ^43^, ^44^, ^45^ ^]^ In the radar chart, six important parameters of ferroelectric single crystals are compared. It is clear from this chart that Nd‐doped PIN–PMN–PT single crystal has major advantages compared to PMN–PT. Compared with PMN–29PT single crystals, the Nd–PIN–PMN–PT single crystal overcomes the trade‐off of improving piezoelectric performance at the expense of the phase‐transition temperature. In contrast to the addition of dopant Nd to improve the piezoelectricity, the introduction of PIN composition can enhance the coercive field and phase transition temperature of the crystal. Table 2 shows that Nd–PIN–PMN–PT had obviously higher *d*_33_, *ε*_33_/*ε*_0_, *k*_33_, *T*_C_, and *E*_C_ than those of the published PMN–29PT. Specifically, *d*_33_ and *ε*_33_/*ε*_0_ of our single crystal were 72% and 51% higher than those of PMN–29PT, respectively. Moreover, *T*_C_ and *E*_C_ of the Nd–PIN–PMN–PT single crystal were ≈34 °C and 37.5% higher, respectively, than those of PMN–29PT. Compared with the undoped ternary single crystal, the doped single crystal has an overwhelming advantage in the dielectric and piezoelectric properties. Compared with Sm‐doped PMN–PT and Nd‐doped PMN–PT, Nd‐doped PIN–PMN–PT had much higher *T*_RT_, *T*_C_, and *E*_C_. Because PMN–PT is the current mature commercial single‐crystal material, the better performance of Nd–PIN–PMN–PT could make it a very powerful competitor in piezoelectric materials.

**Table 2:** Dielectric and piezoelectric properties of Nd–PIN–PMN–PT single crystal and other published relaxor ferroelectric single crystals

Single crystal	*d*_33_ [pC N^−1^]	*ε*_33_/*ε*_0_	*T*_C_ [° C]	*T*_RT_ [° C]	*E*_C_ [kV cm^−1^]	Reference
Nd–PIN–PMN–PT (sample C)	324	9246=	164	91	3.3	This work
PMN–29PT	1881	6140	130	92	2.4	[24]
PIN–PMN–32PT	1497	4372	172	111	4.4	[33]
PIN‐PMN‐PT	1285	4753	155	110	4.5	[43]
23PIN‐PMN‐30PT (ACP)	2000	6200	165	125	3.1	[44]
Sm–PMN–30PT	3600	12 300	115	61	2.6	[11]
Nd–PMN–30PT	3090	12 526	138	87	2.1	[45]

### 2.6 Temperature‐Dependent Piezoelectric and Ferroelectric Properties

**Figure 6:**
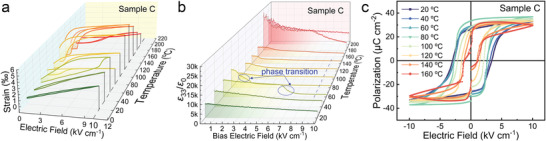
a) Strain, b) dielectric permittivity under bias electric field, and c) hysteresis loop of sample C at different temperatures.

The dielectric, piezoelectric, and ferroelectric properties are usually sensitive to changes in environmental conditions. To evaluate these aspects, the properties of sample C were tested under various electric fields and temperatures (**Figure** **6**). The test temperature range was 20–200 °C, which covered all phasetransition temperatures. In Figure 6a, the strains at different temperatures were measured under a unipolar electric field. At room temperature, the strain was linear with low hysteresis. When the temperature was increased to 40 °C, the strain of sample C still remained linear but the hysteresis became stronger. This indicates that sample C would undergo a phase transition if a larger electric field or higher temperature was applied. At 60 and 80 °C, the unipolar strain showed obvious phase transition to the tetragonal phase. The strain curves at 100–160 °C exhibited the typical profile of the tetragonal phase. In the same temperature range, the slopes of the linear part were relatively low (around 400 pm V^−1^), which was consistent with the piezoelectric response of the tetragonal phase. The changes in the strain curves between 80 and 100 °C indicated a phase transition temperature, corresponding to *T*_RT_ in Figure 5a. In addition, as the temperature increased above 180 °C (above *T*_C_), sample C possessed the cubic rather than tetragonal phase structure. Thus, the strain was mainly from the electrostrictive effect as the cubic phase usually does not exhibit piezoelectricity. Hysteresis loops at different temperatures are plotted in Figure 6c. As the temperature increased, *E*_C_ decreased and the hysteresis loop tended to narrow. The dielectric permittivity under a biased electric field at different temperatures is shown in Figure 6b. The dielectric permittivity decreased with increasing applied bias electric field at room temperature. Moreover, the electric‐field‐induced phase transition occurred more easily near *T*_RT_, as indicated by blue arrows and circles in the figure. The blue dashed line indicates similar values of dielectric permittivity at different temperatures, which implies that they had the same phase structure (tetragonal phase). Figure S7a, Supporting Information, shows the dielectric loss under a bias field at different temperatures. These curves had a similar profile and trend as the dielectric permittivity.

## 3 Conclusion

The rare‐earth dopant Nd^3+^ substituted for A‐site Pb^2+^ ions in relaxor ferroelectric single crystal PIN–PMN–PT. The dopant ions and resulting Pb vacancies intensified the fluctuations in the lattice parameter, which disrupted the long‐range‐ordered domain structure. Rayleigh analysis confirmed that the additional PNRs contributed to intrinsic piezoelectric response and irreversible interface motion, which resulted in single crystals with enhanced piezoelectric and dielectric coefficients (*d*_33_ > 3000 pC N^−1^ for samples B, C, and D) at room temperature. The outstanding piezoelectric and dielectric properties make Nd–PIN–PMN–PT single crystals suitable for high‐frequency acoustic applications such as highly accurate medical transducer arrays. In addition, the segregation of dopant Nd^3+^ was opposite to that of PT along the boule. Because both Nd^3+^ ions and PT positively favor the piezoelectric and dielectric properties, their opposite segregation reduced the variation in the properties along the single crystal boule. As a result, it is estimated that over 75% of the entire single crystal boule was useable volume, which could reduce the cost for final applications compared to undoped crystals obtained by conventional growth methods with much lower available volumes. Furthermore, Nd–PIN–PMN–PT single crystal had a relatively good temperature and electric field stability (164 °C and 3.3 kV cm^−1^, respectively). Compared with commercial PMN–29PT, Nd–PIN–PMN–PT overcomes a typical trade‐off between piezoelectric properties and phase transition temperature/coercive field, which makes it more feasible for complex fabrication processes. Overall, Nd–PIN–PMN–PT single crystals could be a promising competitor among piezoelectric materials for future device design.

## 4 Experimental Section

### Single‐Crystal Growth

The modified vertical Bridgman method was applied to grow Nd–PIN–PMN–PT single crystal.^[^ ^11^ ^]^ The nominal composition was selected to be Nd_0.004_Pb_0.994_[(In_1/2_Nb_1/2_)_0.24_(Mg_1/3_Nb_2/3_)_0.45_Ti_0.31_]O_3_. In order to avoid the pyrochlore phase, a modified Columbite precursor method was used to pre‐synthesize the Columbite precursors, MgNb_2_O_6_, and InNbO_4_, all of which possessed structures similar to that of the perovskite phase. The MgNb_2_O_6_ and InNbO_4_ were synthesized by solid‐state reaction at 1200 and 1100 °C, respectively. Subsequently, the raw materials Nd_2_O_3_, PbO, InNbO_4_, MgNb_2_O_6_, and TiO_2_ were mixed and calcinated at a lower temperature (850–950 °C) to synthesize Nd–PIN–PMN–PT powder with pure perovskite structure, which was confirmed by XRD, as shown in Figure S8, Supporting Information. An Nd–PIN–PMN–PT single‐crystal boule with a diameter of 25.4 mm was grown in the modified Bridgman furnace with two zones.^[^ ^11^ ^]^ The temperature of the upper zone was set to 30–200 °C higher than the melting point of the crystal, while the temperature of the lower zone was set to 50–300 °C lower than the melting point. During crystal growth, after soaking for 12 h, the platinum crucible containing the melted powder was cooled slowly over 20 d by slowly moving the crucible from the upper zone to the lower zone at a speed of 0.41 mm h^−1^. A [001] crystal seed was used to induce unidirectional crystallization. Finally, the crystallinity and direction of the as‐grown single‐crystal boule were confirmed and orientated by XRD.^[^ ^33^ ^]^

### Sample Preparation

Single‐crystal samples of Nd–PIN–PMN–PT were cut into square plates with dimensions of 5 mm × 5 mm × 1 mm, where the largest surface was perpendicular to the [001] direction. To confirm sample uniformity, the single‐crystal samples were cut at every 20 mm length of the crystal boule, and obtained four batches of single‐crystal samples (A, B, C, and D), as shown in Figure 1a. Sample A was cut from the bottom of the boule, while sample D was cut from the top. Every batch contained more than five samples to ensure the reproducibility of the experiment. Gold was vacuum‐sputtered onto the top and bottom surfaces as electrodes for later measurements. An AC field was used with 1500 V mm^−1^ amplitude and 1 Hz frequency, to pole the single‐crystal samples. The samples were polished for more than 5 h with 7‐, 5‐, 3‐, and 0.5‐micron polishing pastes, sequentially. Subsequently, the sample composition was analyzed using EPMA with a JEOL JXA‐8230 superprobe. For XRD measurements, the single‐crystal samples were ground into powder. The piezoelectric coefficient *d*_33_ was measured using a *d*_33_ meter (ZJ‐3A, China). The dielectric permittivity (*ε*_33_/*ε*_0_) and loss (tg*δ*_e_) were measured using two LCR meters (Agilent 4294 and 4980) at cryogenic temperature and high temperature, respectively. The strain and hysteresis loop curves were measured using a ferroelectric measurement system (aixACCT TF 2000 analyzer, Germany).

## Conflict of Interest

The authors declare no conflict of interest.

## Supporting information

Supporting Information

## Data Availability

The data that support the findings of this study are available from the corresponding author upon reasonable request.
