# Supplementary material for: Achieving Giant Piezoelectricity and High Property Uniformity Simultaneously in a Relaxor Ferroelectric Crystal through Rare‐Earth Element Doping
Source: Adv Sci (Weinh). 2022 Oct 26;9(35):2204631. doi: 10.1002/advs.202204631 (PMC9762314; doi:10.1002/advs.202204631)
Supplement: Supplementary file 1 — Supporting Information [file ADVS-9-2204631-s001.pdf]

ADVANCED SCIENCE
Open Access

Supporting Information

for *Adv. Sci.*, DOI 10.1002/advs.202204631

Achieving Giant Piezoelectricity and High Property Uniformity Simultaneously in a Relaxor Ferroelectric Crystal through Rare-Earth Element Doping

Yangbin Liu, Qian Li, Liao Qiao, Zhuo Xu and Fei Li**

Supporting Information

Achieving giant piezoelectricity and high property uniformity simultaneously in a relaxor ferroelectric crystal through rare-earth element doping

Yangbin Liu, Qian Li, Liao Qiao, Zhuo Xu, Fei Li**

Electronic Materials Research Laboratory, Key Laboratory of the Ministry of Education & International Center for Dielectric Research, School of Electronic and Information Engineering, Xi’an Jiaotong University, Xi’an 710049, China

E-mail: ful5@xjtu.edu.cn

E-mail: xuzhuo@xjtu.edu.cn

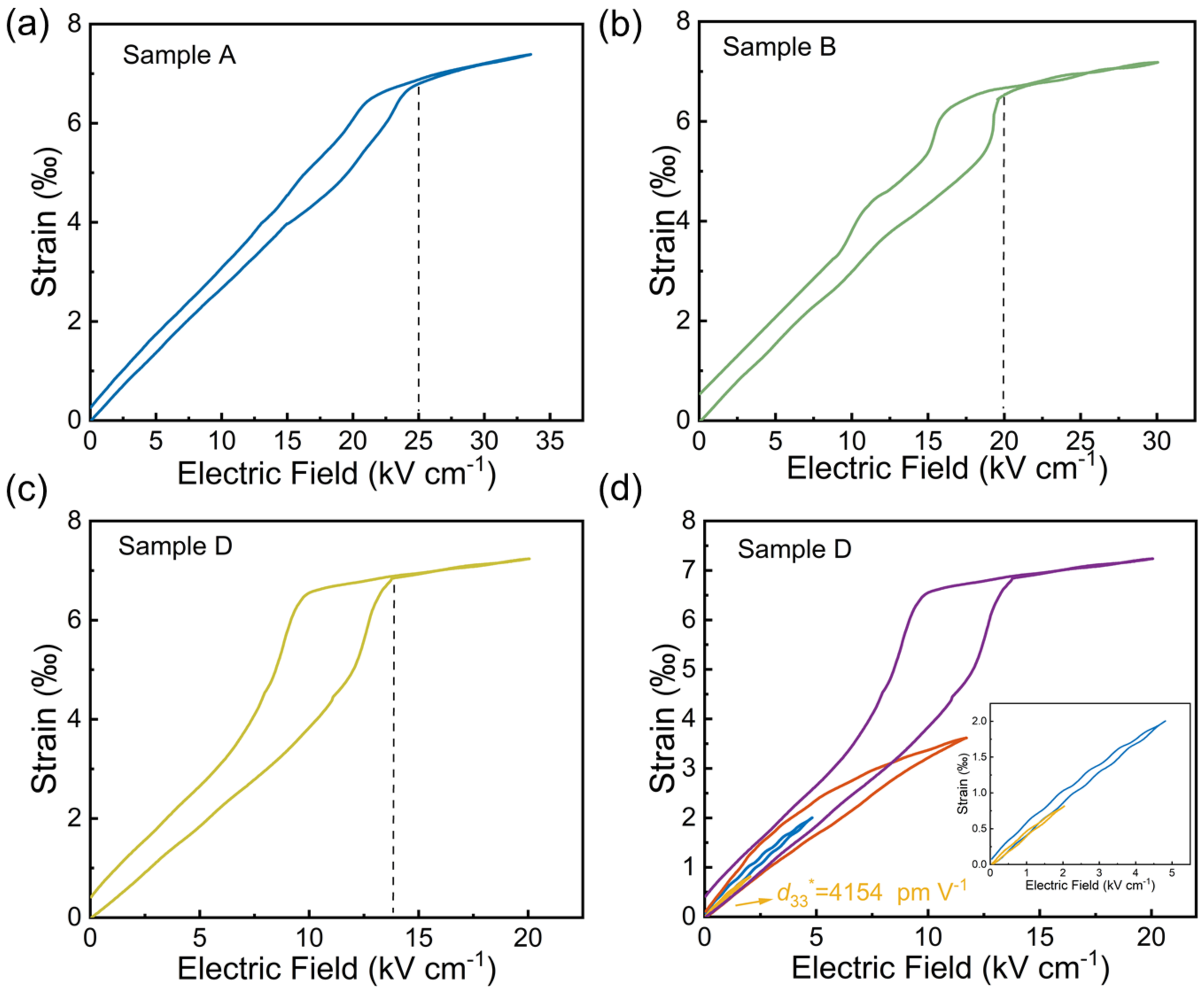

Figure S1. The strain under a high electric field of (a) sample A, (b) sample B, (c) sample D. (d) showed the strain of sample D under various electric fields to details of hysteresis and phase transition.

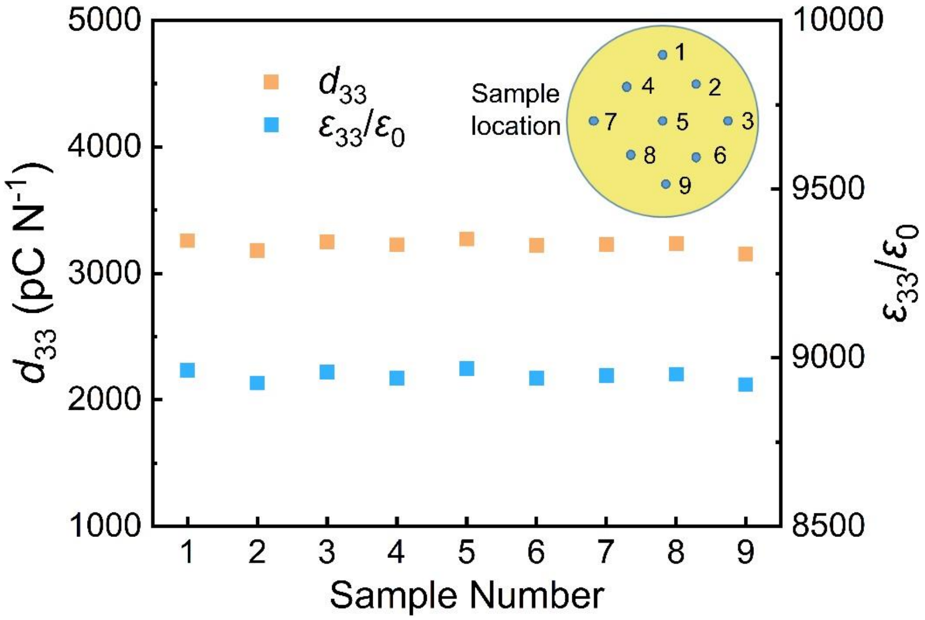

Figure S2. The piezoelectric coefficient d_{33} and dielectric permittivity $\varepsilon_{33}/\varepsilon_0$ were measured at different position in radial direction. The measured postion was pointed and numbered at top-right in the yellow circle. The variations of d_{33} and $\varepsilon_{33}/\varepsilon_0$ in radial direction were less than 5%.

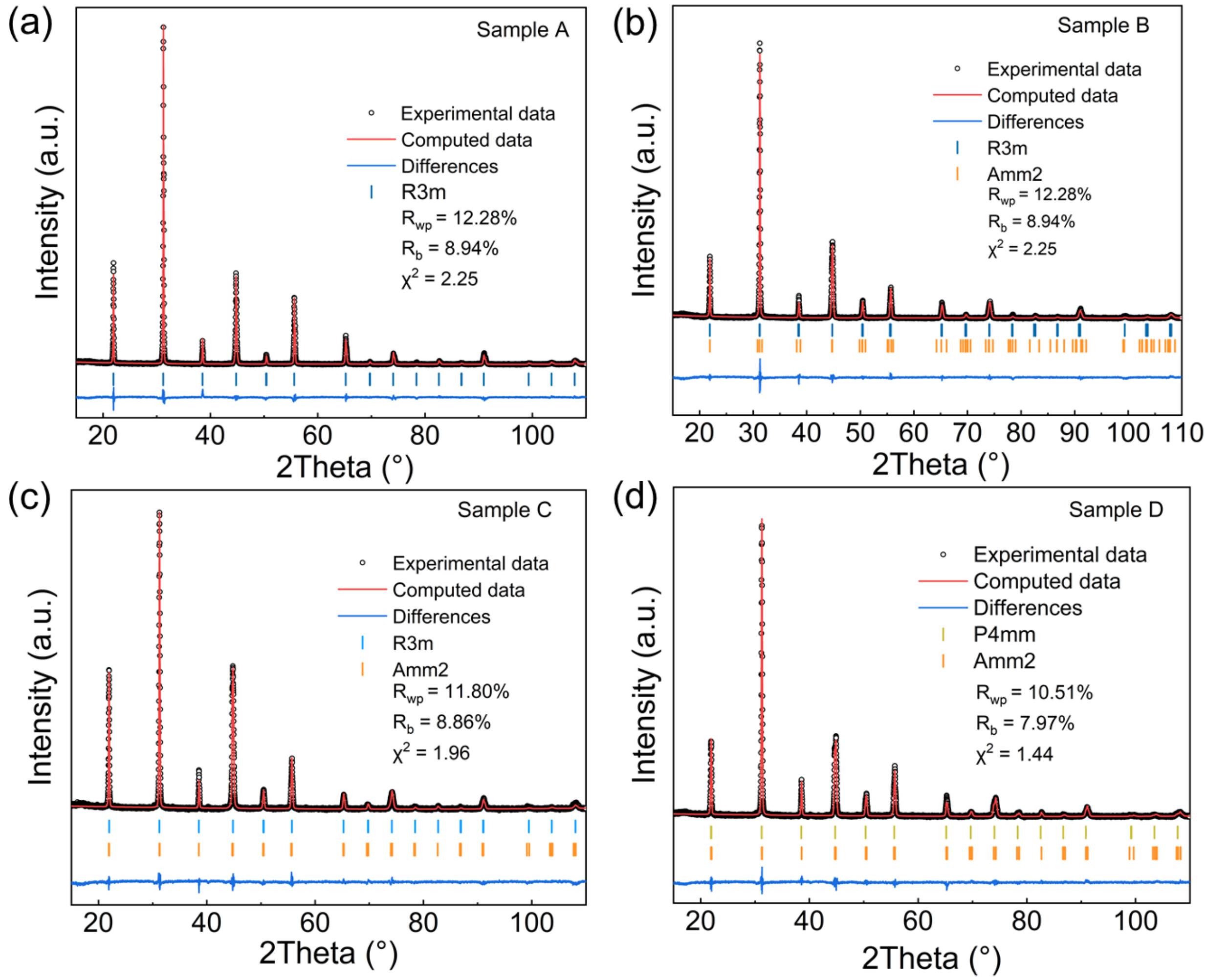

Figure S3. Refinement of XRD patterns by Rietveld method for (a) sample A, (b) sample B, (c) sample C and (d) sample D.

Table S1. The lattice parameters obtained by XRD experiments.

Sample	Space Group	a(Å)	b(Å)	c(Å)	alpha(°)	beta(°)	gama(°)
A	R3m	4.041445	4.041445	4.041445	89.985	89.985	89.985
B	R3m	4.041378	4.041378	4.041378	89.895	89.895	89.895
	Amm2	4.049192	5.7914867	5.6458178	90	90	90
C	R3m	4.037854	4.037854	4.037854	89.986	89.986	89.986
	Amm2	4.0483813	5.706289	5.708241	90	90	90
D	Amm2	4.054424	5.6991673	5.7053576	90	90	90
	P4mm	4.0477843	4.0477843	4.05276	90	90	90

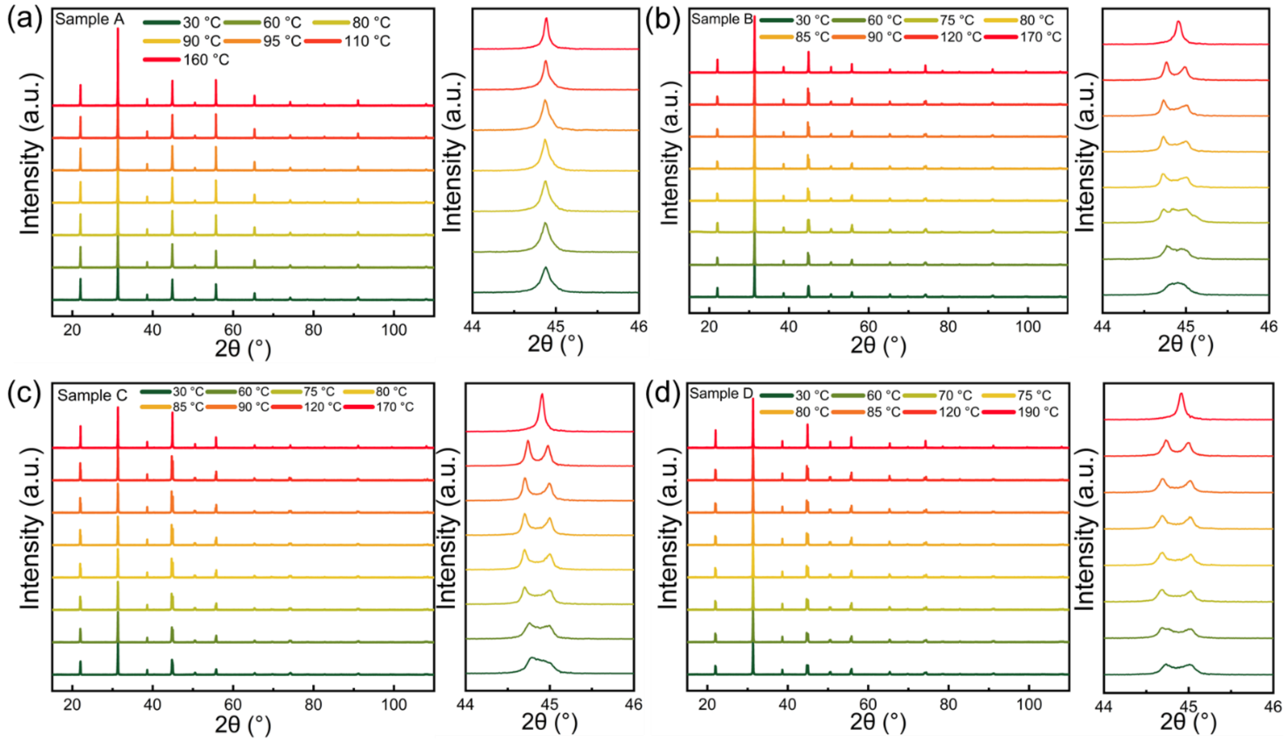

Figure S4. XRD patterns of the powder of the samples. (a) sample A, (b) sample B, (c) sample C, and (d) sample D at various temperatures.

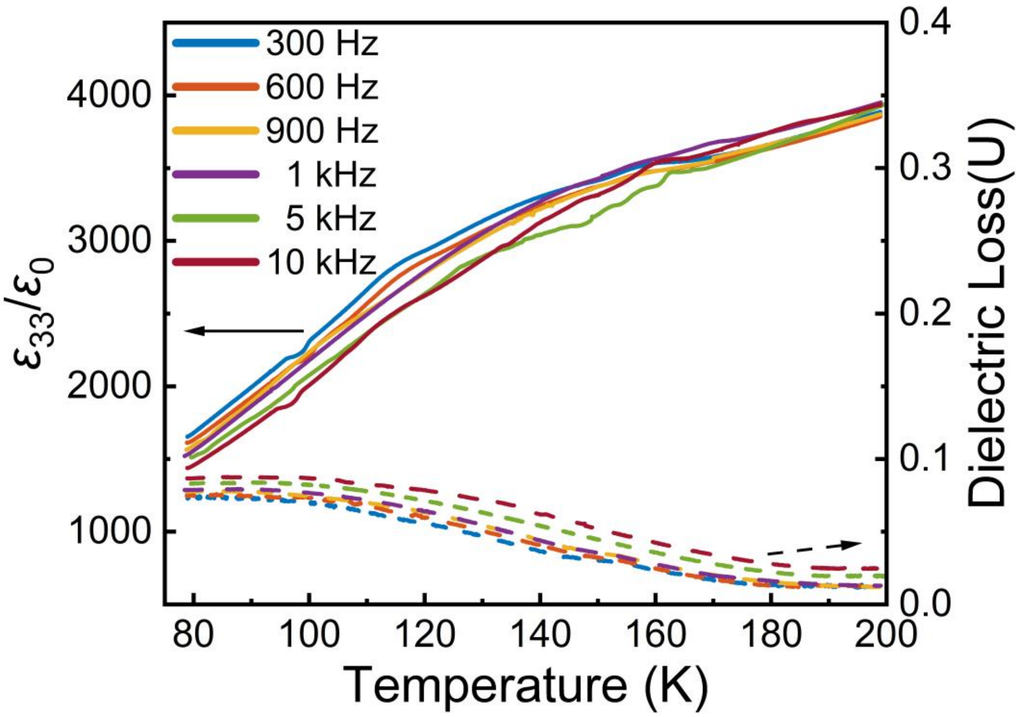

Figure S5. The low-temperature dielectric permittivity at different frequencies(300 Hz- 10 kHz).

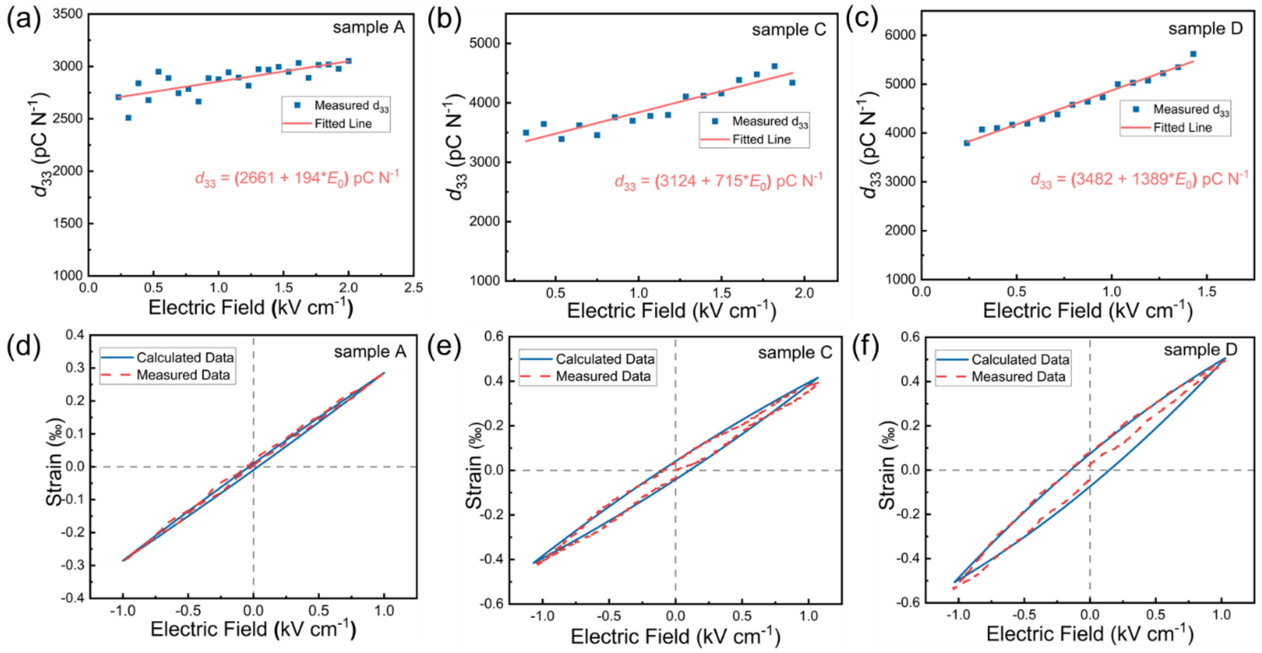

Figure S6. Experimental data and fitted lines of (a–c) d_{33} and (d–f) strain for samples (a,d) A, (b.e) C, and (c.f) D with respect to electric fields.

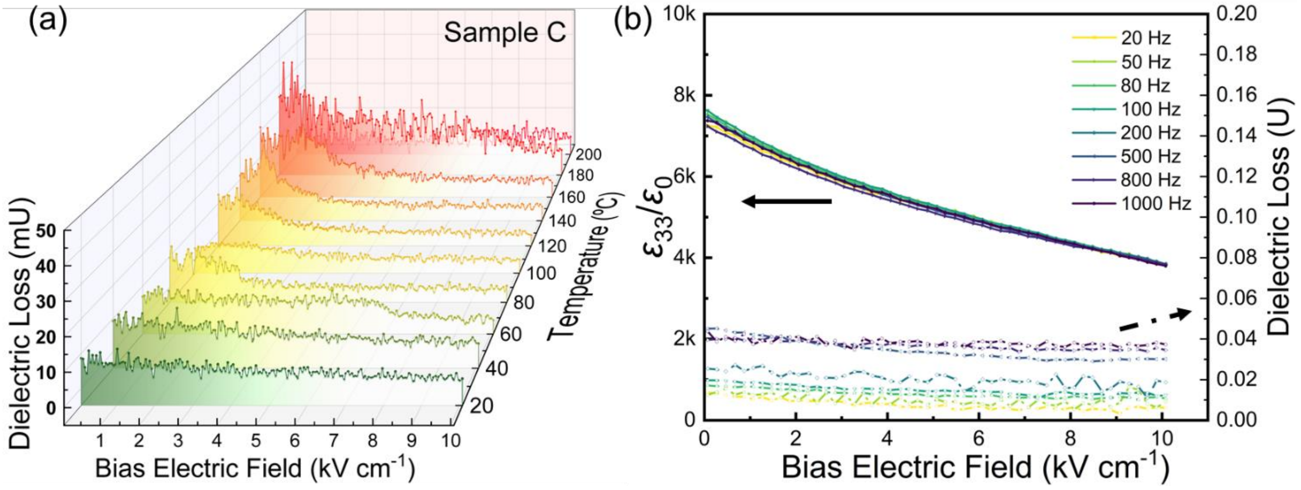

Figure S7. (a) Dielectric loss of sample C under bias electric field at various temperatures showing a similar trend as the dielectric permittivity versus bias electric field. (b) Frequency-dependent dielectric permittivity and loss under bias electric field, where dielectric permittivity is not sensitive to the frequency.

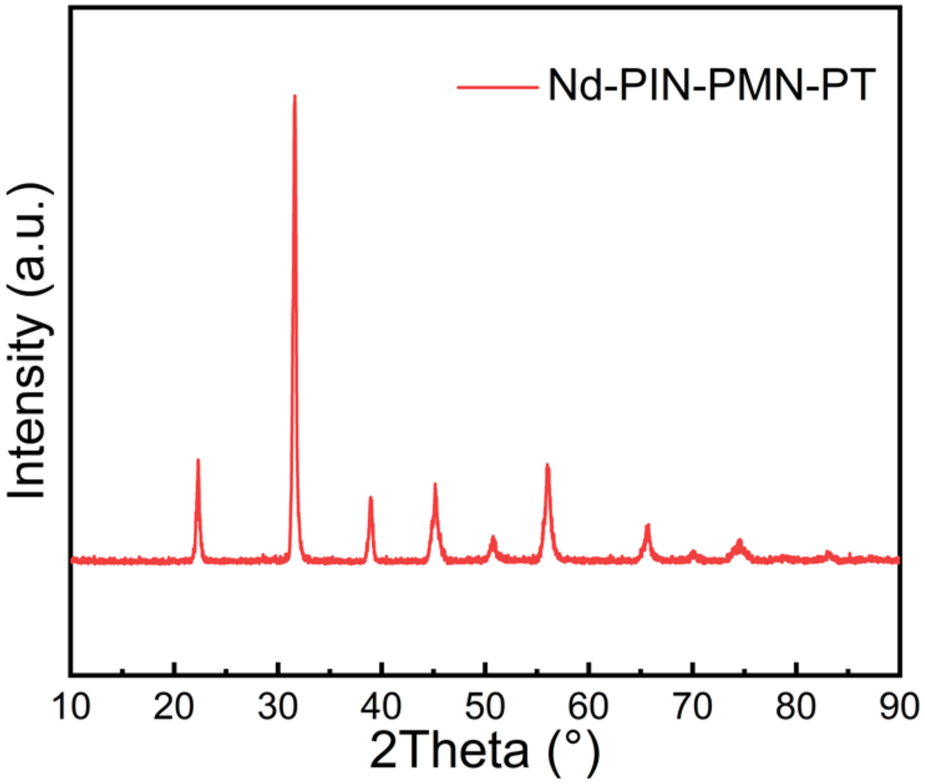

Figure S8. XRD patterns of synthesized $Nd_{0.004}Pb_{0.994}[(In_{1/2}Nb_{1/2})_{0.24}(Mg_{1/3}Nb_{2/3})_{0.45}Ti_{0.31}]O_3$ powder.
